# Ventilator brand, not ventilation mode, determines electricity consumption and estimated carbon emissions: a bench study of nine intensive care ventilators

**DOI:** 10.3389/fmed.2026.1966196

**Published:** 2026-10-21

**Authors:** Semahattin Serkan Sezer, Nimet Şenoğlu

**Affiliations:** Department of Intensive Care, Çiğli Training and Research Hospital, İzmir Bakırçay University, İzmir, Türkiye

**Keywords:** carbon footprint, energy consumption, green procurement, greenhouse gas emissions, intensive care, mechanical ventilation, sustainable health care

## Abstract

**Background:**

Health care generates 4%–5% of global greenhouse gas emissions, and the intensive care unit (ICU) is among its most energy-intensive environments, where energy rather than consumables dominates the carbon footprint. Ventilators run continuously, yet their electricity consumption has rarely been measured directly and never compared between brands.

**Methods:**

Active power was measured with a single plug-in digital meter on three devices from each of three brands, in every available ventilation mode plus standby (90 measurements). The device was the independent experimental unit, giving an effective sample of nine ventilators. All used integral internal gas delivery, with a shared circuit and test balloon under standardised settings. Power was read every 60 s over 10 min per mode and averaged, then modelled by linear mixed effects (brand and harmonised mode as fixed effects, random intercept for device within brand, brand-specific residual variance). The brand effect was corroborated by parametric bootstrap, the mode effect by equivalence testing; a brand-by-mode interaction was fitted. Emissions were projected across several utilisation and grid scenarios.

**Results:**

Estimated marginal means were 64.9 W (Brand A), 95.4 W (Brand B) and 56.3 W (Brand C). Brand exerted a strong effect (bootstrap p=0.003); mode showed no detectable effect under the tested conditions (bootstrap p=0.51), every mode was equivalent to the reference at a pre-defined ±10 W margin, and no brand-by-mode interaction was detected (p=0.27, limited power). Brand B exceeded Brand C by 39.1 W (95% CI 17.0–61.2): a projected 161 kg ${\mathrm{CO}}_{2}$-equivalent (95% CI 70–252) per ventilator annually at the Turkish grid factor under continuous operation, and 17 to 161 kg across scenarios. Standby reached 92.6% of active consumption in one brand; the active-minus-standby increment (4.8, 28.7 and 15.2 W) placed most of the between-brand difference in the idle baseline rather than in ventilation.

**Conclusions:**

Brand, not ventilation mode, dominated electricity consumption among the devices tested. The findings support requiring measured power to be declared and evaluated as a standardised criterion in ventilator tenders; because all devices were approximately ten years old, they do not identify which currently marketed platform is preferable.

## 1 Introduction

Climate change is the defining public health challenge of the century, and health care is not merely a bystander but a substantial driver of the crisis it must confront. A landmark assessment by Health Care Without Harm estimated that health care is responsible for approximately 4.4% of global net greenhouse gas (GHG) emissions—an output so large that, as a nation, the sector would rank as the world’s fifth-largest emitter (1). Independent input–output modelling by Lenzen and colleagues corroborated this magnitude across a spectrum of environmental indicators (2), and earlier national accounting attributed close to 10% of United States GHG emissions to the health sector alone (3). Against a backdrop of successive record-breaking global temperatures, the most recent Lancet Countdown makes clear that decarbonisation across every sector, health included, remains both urgent and unmet (4).

The ICU concentrates this burden. In a life-cycle assessment of septic shock, McGain and colleagues found daily emissions of 178 kg ${\mathrm{CO}}_{2}$-equivalent per patient in a United States ICU and 88 kg in an Australian ICU, and—decisively—that energy for heating, ventilation and air conditioning dominated the footprint, contributing 87% and 76%, respectively, while consumables were comparatively minor (5). A systematic review of the carbon footprint of critical care confirmed this 88 to 178 kg ${\mathrm{CO}}_{2}$-equivalent per patient-day range and again identified energy use as a leading contributor (6). The European Society of Intensive Care Medicine (ESICM) Green Paper has translated this evidence into a call for action, naming energy efficiency and sustainable procurement among its priority domains (7). Together these analyses point to the electrical devices that operate continuously at every ICU bedspace.

The mechanical ventilator is the archetype of such a device: indispensable, in near-constant use and—unlike most consumables—powered without interruption throughout a patient’s respiratory support, frequently for days or weeks. Its relevance is amplified by the scale of its use and the relentless demand for critical care: mechanical ventilation is among the most common interventions in the ICU, concerning essentially all patients at some point in their stay (8), and ICU occupancy remains persistently high, leaving ventilators in continuous, around-the-clock service (9). Despite this, ventilator electricity consumption has seldom been measured directly. The principal exception is the work of Drinhaus and colleagues, who measured the active power of common ICU devices and reported that some equipment, ventilators among them, drew nearly as much power in standby as in active operation; they concluded that switching devices off when not required is both safe and climate-beneficial given their short start-up times (10). Yet even that work did not address the question that most directly informs purchasing policy: whether the choice of ventilator brand itself materially affects consumption and, therefore, emissions.

We are not aware of any prior study that has compared the energy efficiency of different mechanical ventilator brands using multiple devices per brand. Existing analyses have quantified the energy use of ICU equipment as a category, but none has asked whether two devices performing the identical clinical task differ in the electricity—and the emissions—they demand. We address this gap directly. Because the emission consequences of any such difference scale with grid carbon intensity, they are greatest in fossil-dependent systems: in Türkiye, the national grid emits approximately 0.469 kg ${\mathrm{CO}}_{2}$-equivalent per kilowatt-hour delivered to a distribution-connected consumption point (11), so even modest per-device differences may compound into meaningful emissions across a continuously operating fleet. We therefore compared the instantaneous power consumption of three brands of ICU mechanical ventilator, using multiple devices per brand and measuring across every mode offered by each, to determine whether brand or ventilation mode is the stronger driver of electricity use and to express any difference as estimated annual ${\mathrm{CO}}_{2}$ emissions relevant to procurement.

## 2 Materials and methods

### 2.1 Study design and setting

This prospective, observational bench study assessed the instantaneous electrical power consumption of nine ICU mechanical ventilators, comprising three individual devices from each of three commercial brands. The study was conducted at Çiğli Training and Research Hospital, İzmir Bakırçay University, İzmir, Türkiye, at an altitude of 25 m, in an air-conditioned environment with ambient temperature maintained between 20.0 and $22.0{\;}^{\circ }C$.

This was a bench study of medical equipment. It involved no human participants, no animals, no patient data and no identifiable information, and informed consent was therefore not applicable. Institutional approval was nonetheless obtained from the Non-Interventional Clinical Research Ethics Committee of İzmir Bakırçay University (decision no. 2449, research no. 2437, 18 September 2025) in accordance with our institution’s requirements, and the study was conducted in accordance with the principles of the Declaration of Helsinki so far as they apply to non-human research.

Because this was a single-centre study and its findings should be regarded as proof-of-concept—generating a signal that warrants confirmation in larger, multicentre work rather than a definitive ranking—the three brands are reported only as Brand A, Brand B and Brand C, so that no manufacturer is commercially advantaged or disadvantaged on the basis of a preliminary sample. Three considerations support this decision. First, all devices were approximately ten years old, so the platforms measured are unlikely to correspond to the models these manufacturers currently market; naming them could misdirect current purchasing decisions. Second, with three devices per brand the design cannot support a ranking of manufacturers, only the demonstration that clinically equivalent devices can differ. Third, the recommendation this study makes—that measured power consumption be declared and evaluated in tenders—is actionable without identifying any particular manufacturer. The authors have no financial relationship, consultancy arrangement or other conflict of interest with any ventilator manufacturer, and the study received no industry funding; anonymisation reflects scientific caution, not any commercial interest.

### 2.2 Devices

All nine devices were hospital-type (ICU-class) mechanical ventilators of comparable configuration. Critically for the comparison, every device generated its inspiratory flow by means of an integral internal gas-delivery mechanism; none of the brands studied used wall-supplied compressed air as its driving gas. The electrical work of gas delivery was therefore performed by the device itself in every case, and no portion of that work was offloaded to the hospital’s central compressed-air plant. Differences in measured power consumption can consequently be attributed to the devices themselves rather than to a displacement of energy demand to hospital infrastructure.

All nine ventilators were procured for our hospital’s intensive care units within the same procurement period, approximately ten years before the study, and have since been in continuous service in tertiary-level intensive care units. Device age and cumulative clinical exposure were therefore broadly comparable across the three brands, so that age is unlikely to confound the between-brand comparison; the implications of this age for external validity are addressed in the Discussion. All devices were undergoing regular periodic maintenance by the hospital biomedical engineering department and authorised service agents, and none had been referred for repair on account of any fault or malfunction within the preceding year. All devices were thus fully functional and within their normal service life at the time of measurement.

### 2.3 Power consumption measurements

Instantaneous active power (W) was measured using a single plug-in digital power meter (KTEM02-1, KETOTEK; rated 230 V AC, 16 A, maximum load 3680 W; CE-marked; Xiamen Atfinger Technology Co., Ltd., Xiamen, China). The meter reports active power in watts with a display resolution of 0.1 W. According to the manufacturer’s product documentation it provides a power measurement accuracy of ±2%. The unit was calibrated by its manufacturer prior to supply; no additional calibration was performed by the investigators and the meter was not independently verified against a laboratory reference instrument before or after the study, so neither the calibration nor the stated accuracy is independently certified. The meter does not display power factor or reactive power, and these were not recorded; the possibility that the three platforms differ in their power-factor characteristics, and that the meter’s accuracy differs correspondingly between them, therefore cannot be excluded. A single meter unit was used for every measurement, which eliminates inter-meter variability but does not guarantee that measurement error is identical across devices of different electrical design. Each ventilator was connected to the mains supply through the meter.

Before every measurement session the device’s internal battery was verified to be fully charged, and the mains supply was uninterrupted throughout all measurements. No device therefore entered a battery-charging state during data collection, eliminating charging current as a source of between-device variation in measured active power.

For each device, power was measured in every ventilation mode available on that device, plus standby. To capture consumption robustly rather than at an arbitrary instant, the ventilator was allowed to run for 10 min in each mode to reach steady state, during which the displayed active power was read every 60 s; the ten readings were then averaged to yield the value used in analysis. All modes for a given device were measured within a single session, and devices were measured in a fixed order within each brand; measurement order was not randomised. Modes were taken in the order in which they appeared on each device’s interface, which minimised handling and the risk of setting errors over a long session. Because each mode was held for 10 min and devices offered between nine and eleven modes including standby, a complete session ran approximately 90 min for Brand A, 100 min for Brand C and 110 min for Brand B. A formal test for a measurement-order effect is reported in the Results.

### 2.4 Ventilator settings and load

A single shared breathing circuit and rubber test balloon served as the load; the same two physical items were used for every one of the nine devices, so the mechanical load was identical across brands. The balloon was a generic reusable rubber test lung of the type routinely supplied for ventilator function checks. Its manufacturer and model were not recorded at the time of the study and cannot be established retrospectively, and its compliance and resistance were neither specified by the supplier nor measured by us; the consequences of this for reproducibility are addressed in the Limitations. Ventilator settings were standardised across devices as follows, applied in every mode in which the corresponding parameter was adjustable: tidal volume 400 mL, respiratory rate 10 breaths/min, inspiratory-to-expiratory (I:E) ratio 1:2, positive end-expiratory pressure (PEEP) $5\;{\mathrm{cmH}}_{2}O$, inspiratory pressure $15\;{\mathrm{cmH}}_{2}O$ in pressure-controlled breaths, pressure support $10\;{\mathrm{cmH}}_{2}O$ above PEEP and fraction of inspired oxygen (${\mathrm{FiO}}_{2}$) 0.21. Airway pressure release/bilevel ventilation was set with a high pressure of $15\;{\mathrm{cmH}}_{2}O$, a low pressure of $5\;{\mathrm{cmH}}_{2}O$, $T_{\mathrm{high}}$ 2 s and $T_{\mathrm{low}}$ 4 s; this configuration reproduces the same baseline pressure, the same driving pressure, an I:E ratio of 1:2 and 10 cycles per minute as the other modes, so that the applied ventilatory conditions were equivalent across all mode categories. Because the adjustable parameters differ between modes—and a parameter present in one mode may be absent in another—settings were standardised on a per-mode basis rather than globally; the complete per-mode settings are provided in Supplementary Table S1. None of the devices was equipped with adjustable active humidification, removing a further potential source of between-device variability.

Spontaneous and supported modes (pressure support/CPAP) were assessed with the same passive test balloon, without simulated inspiratory effort. Measurements in these modes therefore reflect device behaviour under a passive load—including any apnoea backup ventilation initiated by the device—rather than power consumption during patient-triggered support. This applied identically to all three brands.

### 2.5 Harmonisation of ventilation modes

Because the naming and number of ventilation modes differ between manufacturers, each proprietary mode was mapped to a harmonised mode category (standby, volume control, pressure control, pressure-regulated volume control, pressure support/CPAP, synchronised intermittent mandatory ventilation and airway pressure release/bilevel). Assignment was made on two explicit axes. The first was the breath-control variable: whether inspiration is volume-targeted, pressure-targeted, or dual-targeted with breath-to-breath pressure regulation to a volume target. The second was the breath-initiation pattern: whether breaths are fully mandatory, a mixture of mandatory and spontaneous, or wholly spontaneous with pressure assistance. The full mapping of proprietary mode names to harmonised categories, together with mode-specific power consumption by brand and the number of device-mode measurements contributing to each cell, is provided in Table 4, so that the assignment can be checked for every observation.

Harmonisation is by control logic, not by internal implementation. Two modes assigned to the same category may nonetheless be delivered by different valve control strategies, different turbine or blower speed-regulation schemes, or different inspiratory rise-time algorithms, any of which could carry a distinct electrical cost. The categories therefore capture clinical equivalence, which is the level at which a clinician selects a mode, but not necessarily engineering equivalence; this is a further reason for caution in interpreting the mode effect.

Because manufacturers do not offer identical mode portfolios, the resulting design was unbalanced: two harmonised categories were unavailable on one brand each (pressure-regulated volume control was absent from Brand B, and volume control from Brand C), and the number of proprietary modes mapping to a given category varied between brands. The mixed-effects model accommodates unbalanced data, but contrasts for those two categories are necessarily estimated from two brands rather than three. Only the synchronised intermittent mandatory ventilation category was represented equally across all three brands.

### 2.6 Statistical analysis

Because repeated measurements from the same device across modes are not statistically independent, the device—not the individual measurement—was treated as the unit of analysis. Descriptive statistics are reported as mean ± standard deviation (SD) and median (interquartile range, IQR); normality was assessed with the Shapiro–Wilk test and Q–Q plots. Standby measurements were analysed separately from active modes.

The primary analysis was a linear mixed-effects model (restricted maximum likelihood) with active power as the dependent variable, brand and harmonised mode category as fixed effects, and a random intercept for device nested within brand to account for within-device correlation. Residual variance differed markedly between brands (SD 6.3, 14.1 and 2.9 W for Brands A, B and C; Levene’s test F=16.61, p<0.001), so a homoscedastic residual structure was untenable. The model therefore specified a separate residual variance for each brand, implemented as a varIdent variance function (12). This specification fitted substantially better than the homoscedastic alternative (likelihood-ratio test 27.3 on 2 degrees of freedom, p<0.001) and was adopted as the primary model; results under the original homoscedastic specification are reported alongside it for comparison.

Because between-brand contrasts are estimated at the device level, their denominator degrees of freedom were taken as the number of devices minus the number of brands, which equals six for the primary model and agrees with the Satterthwaite value of 6.1 obtained under the homoscedastic specification. Pairwise comparisons of estimated marginal means were Bonferroni-adjusted. The intraclass correlation coefficient (ICC) was derived from the variance components.

At nine devices, the Satterthwaite approximation is itself unreliable. The brand and mode effects were therefore additionally tested by parametric bootstrap of the likelihood-ratio statistic, with 1,000 simulations under the corresponding null model; this requires no assumption about the reference distribution. A brand-by-mode interaction was fitted to test whether the between-brand difference varied across ventilation modes, and assessed by the same bootstrap procedure with 500 simulations. Because two brand-mode cells are empty by design, the interaction was tested as a cell-means model over the 16 observed combinations against the additive model. Bootstrap p values are Monte Carlo estimates and are reported with their Monte Carlo standard error.

Because a non-significant p value does not establish equivalence, the mode effect was additionally assessed by two one-sided tests (13) against a pre-specified equivalence margin of ±10 W. This margin was chosen because it is approximately one quarter of the between-brand difference and corresponds to under 90 kWh per ventilator per year, a quantity below the threshold at which mode choice would warrant clinical consideration. Results at a tighter margin of ±5 W are also reported.

Because ventilation modes were measured in a fixed, non-randomised order, a linear term for the within-device sequence position was added to the model to test for time-related drift, and Spearman rank correlations between sequence position and power were computed within each brand.

Standby was measured once per device and was therefore not modelled. It is reported descriptively, and the difference between each brand’s active and standby mean is presented as a decomposition of consumption into the power drawn irrespective of ventilation and the increment attributable to ventilation itself; no interval is attached to that decomposition.

A device-level Kruskal–Wallis analysis, which makes no distributional assumption, was reported as a corroborating analysis, with Dunn post hoc comparisons (Bonferroni-adjusted) and the epsilon-squared ($\varepsilon^{2}$) effect size calculated as H/(n−1).

Two sensitivity analyses were performed. The first, planned a priori, repeated the primary model after excluding a single outlying observation. The second was performed post hoc, after inspection of the device-level means revealed that one Brand B device consumed markedly more than its two siblings; the primary model was repeated with that device excluded in order to establish whether the between-brand effect depended on it. Because it was not pre-specified, the second is reported as exploratory and its results are presented alongside, not in place of, those of the primary model.

The original homoscedastic model was fitted in IBM SPSS Statistics, version 27.0.1.0 (14). SPSS MIXED does not provide a group-specific residual variance structure, so the primary brand-heteroscedastic model and all analyses derived from it were fitted in R 4.5.1 (15) using nlme 3.1-168 (16), which implements the variance function of Pinheiro and Bates (12), with estimated marginal means and Bonferroni-adjusted contrasts obtained through emmeans 2.0.3 (17); the parametric bootstrap was implemented in the same script. The two environments return the same point estimates for the homoscedastic model; its confidence limits differ between them by no more than 0.2 W, because R was given the device-level degrees of freedom (6) whereas SPSS applied the Satterthwaite value (6.1). All figures reported here are those of the R implementation, and the complete analysis script and its output are provided as Supplementary Material. A two-sided p<0.05 was considered statistically significant.

### 2.7 Estimation of energy use and carbon emissions

Annual energy use per ventilator was estimated as (Equation 1)

$$E_{\mathrm{annual}}\;(\mathrm{kWh})=\frac{\bar{P}\;(W)\times 8,760\;h}{1,000},$$ (1)

and annual CO_2_-equivalent emissions as (Equation 2)

$$M_{CO_{2}\mathrm{eq}}\;(\mathrm{kg})=E_{\mathrm{annual}}\times 0.469\;\mathrm{kg}\;{\mathrm{CO}}_{2}\mathrm{eq}/\mathrm{kWh},$$ (2)

using the current Turkish grid consumption-point emission factor for distribution-connected users published by the Turkish Ministry of Energy and Natural Resources (11). The consumption-point factor was chosen in preference to the generation-side factor because it incorporates transmission and distribution losses and therefore reflects the emissions attributable to electricity actually delivered to a hospital socket. Confidence intervals for energy and emission estimates were obtained by applying the same linear transformation to the confidence limits of the corresponding power estimates; they therefore reflect uncertainty in measured power alone and do not incorporate uncertainty in the emission factor or in device utilisation. All emission figures are accordingly scenario-based projections under stated assumptions rather than measured quantities. To make the dependence on those assumptions explicit, estimates were additionally computed across three utilisation levels (continuous operation, 75% and 50%) and four grid emission factors spanning fossil-dependent to low-carbon systems (Table 7).

## 3 Results

### 3.1 Descriptive findings

Ninety power measurements were obtained across the nine devices, of which 81 were in active ventilation modes and 9 in standby. Across active modes, mean power was 64.9±6.3 W for Brand A, 95.7±14.1 W for Brand B and 55.9±2.9 W for Brand C (Table 1). Distributions departed from normality in each brand (Shapiro–Wilk p<0.05), driven predominantly by standby measurements and by a single low observation in Brand A—supporting the device-level and non-parametric approaches used alongside the mixed model, and the separate treatment of standby. Brand B exhibited both the highest mean and the widest within-brand spread, largely because one of its three devices consumed systematically more than the other two (device-level means 85.8, 89.4 and 111.9 W; Figure 1); excluding that device reduced the Brand B standard deviation from 14.1 to 6.9 W, bringing it into line with the other brands.

**Table 1:** Power consumption (W) by brand, in active ventilation modes and in standby.

Brand	Measurements (*n*)	Mean ± SD	Median (IQR)	Range	% of active mean	Active—standby (W)
Active ventilation modes						
A	24	64.9±6.3	65.8 (2.8)	36.3–69.4	–	–
B	30	95.7±14.1	93.0 (25.3)	67.2–122.0	–	–
C	27	55.9±2.9	55.4 (2.7)	52.8–66.3	–	–
Standby						
A	3	60.1±5.1	57.7	56.7–66.0	92.6${}^{a}$	4.8${}^{a}$
B	3	67.0±1.0	67.4	65.8–67.7	70.0	28.7
C	3	40.7±1.6	40.9	39.1–42.2	72.9	15.2

IQR, interquartile range; SD, standard deviation. Three devices contributed to every row. Standby measurements were analysed separately from active modes and are excluded from all models of active power. The penultimate column expresses each brand’s standby consumption as a percentage of its own active mean; the final column gives the difference between the two, that is the incremental power attributable to ventilation itself. Standby was measured once per device, so neither quantity carries an interval.

${}^{a}$Calculated against the Brand A active mean of 64.9 W, which includes the single low observation of 36.3 W discussed in the Results; excluding that observation the active mean is 66.2 W, standby represents 90.8% and the increment is 6.1 W.

**Figure 1:**
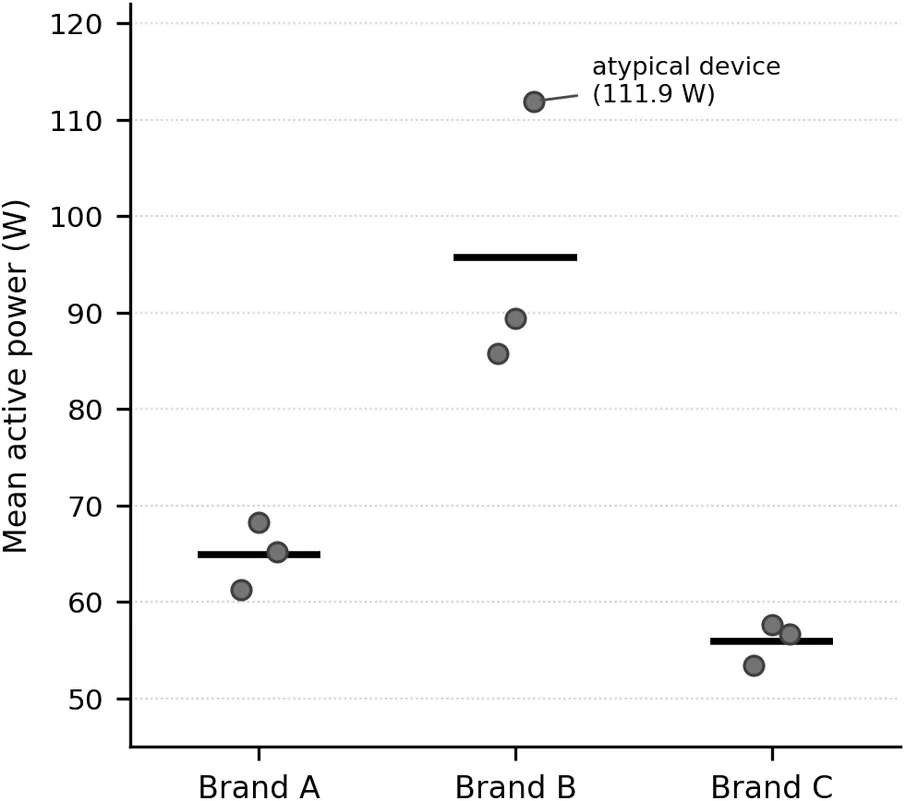
Power consumption by ventilator brand. Individual points show the mean of all active-mode measurements for each of the nine devices (n=3 devices per brand); horizontal bars indicate the brand mean. One Brand B device (111.9 W) consumed substantially more than its two siblings; the post hoc sensitivity analysis excluding this device is reported in the Results.

### 3.2 Standby consumption

Standby power was substantial and differed between brands, mirroring the pattern seen in active operation (Table 1). Mean standby consumption was 60.1±5.1 W for Brand A, 67.0±1.0 W for Brand B and 40.7±1.6 W for Brand C. A device-level Kruskal–Wallis test across brands was significant (H(2)=6.49,p=0.039), although with only three devices per group this test operates close to its attainable minimum p value and the associated effect size is uninformative at this sample size; the substantive finding here rests on the descriptive comparison rather than on the inferential test.

Expressed as a proportion of each brand’s own active consumption, standby represented 92.6% for Brand A, 70.0% for Brand B and 72.9% for Brand C. An idle Brand A device therefore drew almost as much electricity as a fully operating one, closely reproducing the pattern reported by Drinhaus and colleagues (10). Between brands, Brand B in standby consumed more power than Brand C did during active ventilation (67.0 W vs. 55.9 W); 26 of 27 Brand C active measurements fell below the lowest Brand B standby reading. An idle device of the least efficient brand can thus draw more electricity than a fully operating device of the most efficient one. Both observations indicate that standby is not a low-power state in any of the devices studied, and reinforce the practical value of powering ventilators down, rather than leaving them idle, when they are not in use.

Because a device in standby is energised but not ventilating, the difference between a brand’s active and standby mean separates the power drawn irrespective of ventilation from the incremental cost of delivering it. That increment was 4.8 W for Brand A (64.9 vs. 60.1 W; 6.1 W with the single 36.3 W observation excluded), 28.7 W for Brand B (95.7 vs. 67.0 W) and 15.2 W for Brand C (55.9 vs. 40.7 W). Of the 39.8 W separating the Brand B and Brand C active means (the descriptive difference between raw means; the model-based contrast reported below is 39.1 W), approximately 26 W was accordingly already present with both devices idle and approximately 14 W arose from ventilation itself. These are descriptive quantities derived from brand means, with standby measured once per device and no interval attached; they are reported because they locate the greater part of the between-brand difference in subsystems that draw power whether or not the device is ventilating.

### 3.3 Primary analysis

Allowing the residual variance to differ by brand yielded fitted residual standard deviations of 5.61, 8.13 and 2.37 W for Brands A, B and C. In this model, brand was associated with a strong effect on active power (F(2,6)=18.4,p=0.003; parametric bootstrap likelihood-ratio test 17.73 on 2 degrees of freedom, p=0.003, Monte Carlo SE 0.002; F(2,6)=17.1,p=0.003 under the original homoscedastic specification). Estimated marginal mean power was 64.9 W (95% CI 53.2–76.5) for Brand A, 95.4 W (95% CI 83.5–107.3) for Brand B and 56.3 W (95% CI 44.9–67.7) for Brand C. Bonferroni-adjusted comparisons showed that Brand B consumed more than Brand A (difference 30.5 W; 95% CI 8.2–52.9; p=0.012) and Brand C (39.1 W; 95% CI 17.0–61.2; p=0.003), while Brands A and C were not distinguishable (8.6 W; 95% CI −13.3 to 30.4; p=0.73) (Table 2). The pattern is therefore one brand standing apart from two similar ones rather than a graded ranking across three.

**Table 2:** Between-brand comparisons of estimated marginal mean power consumption (linear mixed-effects model, Bonferroni-adjusted), under the primary brand-heteroscedastic specification and under the homoscedastic alternative.

	Primary model (brand-specific variance)			Homoscedastic model		
Comparison	Difference (W)	95% CI	p	Difference (W)	95% CI	p
B vs. A	30.5	8.2 to 52.9	0.012	30.7	7.6 to 53.7	0.014
B vs. C	39.1	17.0 to 61.2	0.003	39.0	15.9 to 62.1	0.004
A vs. C	8.6	−13.3 to 30.4	0.73	8.3	−14.7 to 31.4	0.84

CI, confidence interval. Denominator degrees of freedom=6 (nine devices, three brands). The two specifications agree closely, indicating that modelling the between-brand variance heterogeneity removes the assumption violation without altering the estimates. The primary model fitted substantially better (likelihood-ratio test 27.3 on 2 degrees of freedom, p<0.001). Both columns were fitted in R on 6 degrees of freedom; the same homoscedastic model fitted in SPSS on the Satterthwaite value of 6.1 returns identical point estimates and confidence limits differing by no more than 0.2 W.

The estimates are essentially unchanged from the homoscedastic specification reported in Table 2, so modelling the variance heterogeneity removed the assumption violation without altering any conclusion. Diagnostics improved correspondingly: the Shapiro–Wilk statistic on normalised residuals rose from 0.801 to 0.852, and to 0.938 once the single 36.3 W observation was excluded, indicating that the residual non-normality was attributable to that one reading rather than to the model.

No statistically detectable effect of harmonised mode category on power consumption was observed under the tested conditions (F(5,67)=0.95,p=0.46; likelihood-ratio test 4.88 on 5 degrees of freedom, bootstrap p=0.51, Monte Carlo SE 0.016; F(5,67)=1.04, p=0.40 under the homoscedastic specification). Because a null result does not by itself establish equivalence, the mode effect was assessed against a pre-specified margin. Every harmonised category was statistically equivalent to the reference category at ±10 W (all two one-sided test p<0.001; Table 3). At the tighter margin of ±5 W, equivalence was established for four of the five categories; volume control was the exception (p=0.098), reflecting that this category was available on only two of the three brands. One category, airway pressure release/bilevel, had a 90% interval that excluded zero (−2.13 W, −4.05 to −0.21), so a small difference from the reference cannot be ruled out for it; the coefficient nonetheless lies well inside both equivalence margins and is influenced by the single retained 36.3 W Brand A reading in that category (Table 4). Mode selection therefore carries at most a minor energy penalty relative to the choice of device, bounded at ±10 W under the conditions tested. Mode-specific means by brand are provided in Table 4.

**Table 3:** Equivalence analysis for harmonised ventilation mode category (two one-sided tests against a pre-specified margin), relative to the reference category (SIMV).

Mode category	Coefficient (W)	90% CI	TOST p (±10 W)	TOST p (±5 W)
APRV/bilevel	−2.13	−4.05 to −0.21	<0.001	0.008
Pressure control (PC)	0.30	−1.99 to 2.59	<0.001	<0.001
PRVC	−0.58	−2.99 to 1.83	<0.001	0.002
Pressure support/CPAP	−0.70	−2.60 to 1.19	<0.001	<0.001
Volume control (VC)	1.62	−2.70 to 5.94	<0.001	0.098

CI, confidence interval; TOST, two one-sided tests. Coefficients are from the primary brand-heteroscedastic mixed-effects model. Equivalence is established when the 90% confidence interval lies entirely within the margin. All five categories are equivalent at ±10 W. At ±5 W, volume control does not reach equivalence, reflecting that this category was available on only two of the three brands. The interval for APRV/bilevel excludes zero, so a small difference from the reference cannot be ruled out for that category; the coefficient is nonetheless well inside both margins and is influenced by the single retained 36.3 W Brand A reading in it (Table 4). APRV, airway pressure release ventilation; CPAP, continuous positive airway pressure; PRVC, pressure-regulated volume control; SIMV, synchronised intermittent mandatory ventilation.

**Table 4:** Mode mapping and power consumption (W) by harmonised mode category and brand.

Mode category	Proprietary mode names encountered	Brand A	Brand B	Brand C
Volume control (VC)	VC AC; VC CMV; Volume control	66.2±1.7 (3)	97.8±15.5 (6)	n.a.
Pressure control (PC)	PACV; PC AC; PC CMV; Pressure control	66.2±2.5 (3)	97.3±14.6 (6)	56.5±2.2 (3)
PRVC	PRVC; VACV (PRVC)	65.4±1.0 (3)	n.a.	55.8±2.1 (3)
Pressure support/CPAP	PPSV; PS/CPAP; SPN CPAP/PS; SPN PPS; SPN PS	67.4±2.0 (3)	89.3±16.1 (6)	56.1±3.2 (6)
SIMV	PC SIMV; PSIMV; SIMV PRVC; SIMV Psup; SIMV Vsup; V SIMV (PRVC) + PS; V SIMV + PS; VC SIMV	66.0±2.0 (9)	96.5±13.1 (9)	56.3±4.0 (9)
APRV/bilevel	APRV; P Bilevel; PC BiPAP	56.2±17.3 (3)${}^{a}$	98.5±16.0 (3)	54.8±1.4 (6)

Values are mean ± SD (n), where n denotes the number of device-mode measurements contributing. n.a., mode not available on that brand. Mode portfolios differ between manufacturers, so two categories were available on only two of the three brands; only the SIMV category was represented equally (n=9) across all three.

${}^{a}$Inflated by the single outlying observation of 36.3 W discussed in the Results; excluding it, the Brand A mean for this category is 66.2±1.4 W (n=2). APRV, airway pressure release ventilation; CPAP, continuous positive airway pressure; PRVC, pressure-regulated volume control; SIMV, synchronised intermittent mandatory ventilation.

Fitting a brand-by-mode interaction gave no evidence that the between-brand difference varied across ventilation modes (likelihood-ratio test 12.13 on 8 degrees of freedom, bootstrap p=0.27, Monte Carlo SE 0.020). Two of the ten possible interaction terms are not estimable, because pressure-regulated volume control was unavailable on Brand B and volume control on Brand C, so this assessment is complete only for the four categories present on all three brands. Given nine devices and an unbalanced design the test has limited power, and it should be read as finding no evidence of an interaction rather than as establishing its absence.

Adding the within-device sequence position to the model revealed a small negative trend of −0.45 W per position (95% CI −0.80 to −0.10; likelihood-ratio test 6.65 on 1 degree of freedom, p=0.010), corresponding to a drift of approximately 4 W across the full sequence of modes measured for a device, or 1 to 7 W at the confidence limits. Within individual brands, rank correlations between sequence position and power were not significant (Spearman ρ −0.12, −0.03 and −0.17 for Brands A, B and C; all p>0.38). Because the order was fixed, sequence position and mode category are substantially confounded and the two cannot be separated in these data. Whatever its origin, the magnitude is an order of magnitude smaller than the between-brand difference, and it cannot generate that difference, since the identical fixed-order procedure was applied to every device of every brand.

The between-device variance component (63.8) exceeded the mean residual variance, yielding an ICC of approximately 0.65 (69.6 and 36.0, ICC 0.66, under the homoscedastic specification). As shown below, this clustering was almost entirely attributable to a single device rather than reflecting generalised device-to-device heterogeneity.

### 3.4 Supporting and sensitivity analyses

The device-level Kruskal–Wallis test confirmed a between-brand difference (H(2)=7.20,p=0.027; $\varepsilon^{2}=0.90$). In Dunn comparisons, only the Brand B vs. Brand C contrast survived Bonferroni adjustment (p=0.022)—an expected consequence of three devices per group and the limited resolution of rank-based tests at this sample size; the mixed model, which uses all within-device measurements, retained the power to detect the B vs. A difference.

#### 3.4.1 Exclusion of a single outlying observation (a priori)

One measurement (Brand A, airway pressure release/bilevel, 36.3 W) fell approximately 28 W below every other reading from the same device and could not be attributed to any documented change in device state; we cannot exclude a transient device condition or a recording error. It was retained in the primary analysis and excluded in sensitivity analysis, which altered no conclusion: brand remained significant (F(2,6)=20.1,p=0.002), and both the B vs. A and B vs. C differences persisted (29.4 W, p=0.011 and 39.6 W, p=0.003, respectively). Excluding it reduced the fitted residual standard deviation for Brand A from 5.61 to 1.13 W, confirming that the residual non-normality noted above was attributable to this single reading rather than to the model. Its occurrence nonetheless indicates that single readings can deviate materially, and supports the use of averaged repeated measurements rather than instantaneous values.

#### 3.4.2 Exclusion of a single outlying device (post hoc)

This analysis was decided upon after inspection of the device-level means and forms no part of the pre-specified analysis plan; it is reported as exploratory, and the results of the primary model are presented alongside it in Table 5 rather than being superseded by it.

**Table 5:** Primary and post hoc analyses presented side by side.

Quantity	Primary model (all nine devices)	Post hoc (atypical device excluded)
B vs. A	30.5 W (8.2–52.9), p=0.012	22.5 W (12.3–32.6), p=0.002
B vs. C	39.1 W (17.0–61.2), p=0.003	31.2 W (21.6–40.8), p<0.001
A vs. C	8.6 W (−13.3 to 30.4), p=0.73	8.7 W (0.7–16.8), p=0.037
Device variance component	63.8	5.3
ICC	0.65	0.16
Devices contributing	9	8

ICC, intraclass correlation coefficient. Values are mean differences with 95% confidence intervals, Bonferroni-adjusted, on 6 and 5 denominator degrees of freedom respectively. The post hoc analysis was decided upon after inspection of device-level means and forms no part of the pre-specified analysis plan; it is reported as exploratory. The Brand A versus Brand C comparison is exploratory in both columns and should not be interpreted as establishing a difference between those brands.

Excluding the Brand B device whose consumption exceeded that of its two siblings by approximately 25 W across all modes, the between-brand effect persisted and became more precisely estimated: Brand B exceeded Brand A by 22.5 W (95% CI 12.3–32.6, p=0.002) and Brand C by 31.2 W (95% CI 21.6–40.8, p<0.001), the latter corresponding to approximately 273 kWh and 128 kg ${\mathrm{CO}}_{2}$-equivalent per ventilator per year. The device-level variance component fell from 63.8 to 5.3 and the ICC from 0.65 to 0.16, indicating that essentially all of the within-brand device clustering observed in the primary model was attributable to this single unit, and that the remaining devices were highly homogeneous within their brands. In this reduced model the Brand A vs. Brand C difference also reached nominal significance (8.7 W, 95% CI 0.7–16.8, p=0.037); its lower confidence limit lies close to zero, and because the comparison was not pre-specified it should not be interpreted as establishing a difference between Brands A and C. The headline between-brand finding is therefore not dependent on the atypical device: excluding it attenuates the magnitude of the difference while increasing the precision with which it is estimated.

Three interpretations of this device were considered. Ordinary device-to-device variation is unlikely, since excluding it reduces the device-level variance component more than tenfold and its deviation is comparable in magnitude to the between-brand difference itself. A spurious measurement is also unlikely, since the elevation was consistent across all ten of its modes rather than confined to isolated readings. The most plausible remaining explanation is an unidentified technical condition, which we could not investigate because the device was in clinical service and no internal instrumentation was performed.

The standby measurements narrow that possibility usefully. The three Brand B devices lay within 1.9 W of one another in standby (range 65.8 to 67.7 W), so the atypical unit drew essentially the same idle power as its siblings while exceeding them by approximately 25 W in every active mode. Its excess was thus confined to periods when the device was ventilating, which argues against the continuously energised subsystems—internal power-supply losses, battery float-charging, display and cooling—and points instead to the gas-delivery path, for example increased mechanical resistance or a degraded blower or valve. This inference rests on a single standby measurement per device and is offered as a direction for investigation rather than as a diagnosis.

### 3.5 Estimated energy use and emissions

The following figures are projections under stated assumptions, not measured quantities. Assuming continuous operation, estimated annual energy use per ventilator was approximately 568 kWh (95% CI 466–670) for Brand A, 836 kWh (95% CI 732–940) for Brand B and 493 kWh (95% CI 393–593) for Brand C, corresponding to approximately 267, 392 and 231 kg ${\mathrm{CO}}_{2}$-equivalent per ventilator per year at the Turkish grid factor (Table 6).

**Table 6:** Estimated annual energy use and ${\mathrm{CO}}_{2}$-equivalent emissions per ventilator (continuous operation; Turkish grid consumption-point factor 0.469 kg ${\mathrm{CO}}_{2}$-eq/kWh).

Brand	Marginal mean power, W (95% CI)	Annual energy, kWh (95% CI)	Annual ${\mathrm{CO}}_{2}$-eq, kg (95% CI)
A	64.9 (53.2–76.5)	568 (466–670)	267 (219–314)
B	95.4 (83.5–107.3)	836 (732–940)	392 (343–441)
C	56.3 (44.9–67.7)	493 (393–593)	231 (184–278)

CI, confidence interval. Estimates derive from the primary brand-heteroscedastic mixed-effects model. Intervals are confidence intervals for the brand mean, not prediction intervals for an individual device. They propagate uncertainty in measured power only, and do not incorporate uncertainty in the grid emission factor or in device utilisation. All figures are scenario-based projections under an assumption of continuous operation, not measured quantities; see Table 7.

**Table 7:** Scenario sensitivity of the projected annual ${\mathrm{CO}}_{2}$-equivalent difference between Brand B and Brand C (39.1 W), per ventilator, across utilisation levels and grid emission factors.

Utilisation	0.469 kg/kWh	0.44 kg/kWh	0.25 kg/kWh	0.10 kg/kWh
	(Türkiye)	(global average)	(EU average)	(low-carbon)
Continuous (8760 h)	161 (70–252)	151 (66–236)	86 (37–134)	34 (15–54)
75% (6570 h)	121 (52–189)	113 (49–177)	64 (28–101)	26 (11–40)
50% (4380 h)	80 (35–126)	75 (33–118)	43 (19–67)	17 (7–27)

Values are kilograms of ${\mathrm{CO}}_{2}$-equivalent per ventilator per year, with 95% confidence intervals derived from the confidence limits of the power difference. Across the range examined the projected difference varies roughly tenfold, from 17 to 161 kg. The direction and relative ordering of brands are unaffected by these assumptions.

The 39.1 W difference between the most consumptive (Brand B) and least consumptive (Brand C) brands equates to approximately 343 kWh (95% CI 149–537) and 161 kg ${\mathrm{CO}}_{2}$-equivalent (95% CI 70–252) per ventilator annually; the corresponding figure for Brand B vs. Brand A is 125 kg (95% CI 34–217). These intervals are wide, and the lower bounds are considerably smaller than the point estimates; the direction of the difference is well supported, but its magnitude should be regarded as imprecisely estimated. Expressed proportionally, the B vs. C difference represents 41.0% of Brand B’s own consumption; equivalently, Brand B consumed 69.5% more power than Brand C.

The absolute magnitude depends heavily on the assumptions adopted. Across three utilisation levels and four grid emission factors, the projected Brand B vs. Brand C difference ranges from 17 to 161 kg ${\mathrm{CO}}_{2}$-equivalent per ventilator per year, a roughly tenfold span (Table 7). The direction and relative ordering of the brands are unaffected by these choices, and it is on the latter that the argument of this paper rests.

Scaled to a 20-bed ICU, selecting the more efficient brand would correspond to approximately 3.2 tonnes of ${\mathrm{CO}}_{2}$-equivalent per year (95% CI 1.4–5.0) avoided from ventilator electricity alone. This figure assumes every bed ventilated continuously throughout the year and should therefore be read as an upper bound; at a more realistic 75% ventilated-bed occupancy it falls to approximately 2.4 tonnes (95% CI 1.0–3.8). Neither figure accounts for any additional cooling load these devices impose on the unit’s air-conditioning system, which would act in the opposite direction.

## 4 Discussion

In this comparative bench study of nine ICU mechanical ventilators, the brand of ventilator—rather than the ventilation mode—was the dominant measured determinant of electricity consumption. After accounting for within-device correlation and controlling for mode, the least efficient brand consumed approximately 22 to 39 W more than its comparators, depending on the comparator brand and on whether a single atypical device was included. This difference was statistically robust within the sample and, projected over continuous operation, potentially consequential. The finding that mode exerted at most a minor effect is equally notable: under the bench conditions tested, every mode category was equivalent to the reference within ±10 W, so mode selection carried no appreciable energy penalty and the energy consequence resided almost entirely in the upstream procurement decision. This bound applies to the conditions actually tested. Spontaneous and supported modes were assessed against a passive load without simulated inspiratory effort, and triggering, demand-responsive flow delivery and cycling all impose work that our measurements did not capture; the equivalence bound therefore cannot be extended to patient-triggered ventilation during genuine clinical use.

We are not aware of a previous study comparing energy efficiency between mechanical ventilator brands using multiple devices per brand. Drinhaus and colleagues demonstrated that ventilators and monitors can draw nearly as much power in standby as in operation, and that powering devices down when unneeded is a safe emission-reduction measure (10); our standby findings independently reproduce that pattern, and do so strikingly for one brand, whose idle consumption reached 92.6% of its active consumption. The central contribution here, however, is different: even among devices performing an identical clinical function under identical ventilatory conditions, intrinsic energy efficiency appeared to vary substantially between manufacturers. Placed within the established finding that energy, not consumables, dominates the ICU footprint (5, 6), and within the ESICM Green Paper’s explicit call for energy-efficient, sustainable procurement (7), this suggests that the energy performance of candidate devices deserves explicit consideration at the point of purchase, consistent with the central tenet of the field: that meaningful emission reductions are frequently attainable without compromising the safety or efficacy of care (18, 19).

### 4.1 Scale, permanence and device ageing

The potential importance of the between-brand difference lies in its scalability and its permanence. A difference of this order per ventilator per year would be fixed at the moment of purchase and would recur for the full service life of each device. Its magnitude emerges only when scaled. Ventilators are among the most continuously operated device classes in any hospital, given that mechanical ventilation concerns essentially all ICU patients at some point (8) and that ICU occupancy runs persistently high (9). A single tertiary hospital may run dozens of ventilators around the clock, and national health systems operate substantially larger fleets; although no authoritative inventory of the installed base is available for Türkiye or globally, the aggregate is plainly large, and pandemic-era procurement expanded it further. At any such scale, a per-device difference of this order would aggregate into a considerable annual total, all of it potentially avoidable through purchasing choices that impose no clinical trade-off. Because the emission impact scales directly with grid carbon intensity, the benefit would be greatest precisely in fossil-dependent systems such as Türkiye’s (11), where it is also most easily overlooked. As health systems worldwide move toward mandatory emissions reporting and net-zero commitments (20), the energy performance of continuously operating equipment deserves consideration alongside the more familiar targets of anaesthetic gases, single-use consumables and waste (19).

What might account for one platform consuming substantially more than the others? Our measurements allow one step beyond speculation. Because a device in standby is energised but not ventilating, the active-minus-standby increment separates the continuously energised baseline from the electrical cost of ventilation itself, and that decomposition places approximately two-thirds of the Brand B vs. Brand C difference in the baseline rather than in gas delivery. Attention is therefore directed first at subsystems that run irrespective of ventilation: the efficiency of the internal power supply, and in particular whether it uses an older linear or a switched-mode design; continuous cooling fans; display technology and backlight; anti-condensation heating of expiratory valves or flow sensors; and float-charging circuitry for the internal battery, which continues to draw current even when the battery is fully charged, as was verified before each session here. The remaining third is attributable to gas delivery, where the architecture also differs between platforms: a turbine or blower held at high speed with flow modulated by a proportional valve imposes a different demand from one that accelerates and decelerates on a per-breath basis, and from a piston or internal compressor recharging on a duty cycle. We did not instrument the devices internally, so these remain hypotheses rather than findings; what the decomposition establishes is where to look, not which component is responsible.

The same decomposition offers a reading of the null mode effect. If the incremental power required to ventilate is only 5 to 29 W depending on the platform, then any difference between ventilation modes must be a fraction of that increment, and the ±10 W equivalence bound established here is consistent with a device whose consumption is dominated by what it does continuously rather than by the breath pattern it delivers. This also implies that the practical lever available to clinicians lies in whether a device is running at all, not in how it is set.

A second observation warrants attention. One Brand B device consumed approximately 25 W more than its two siblings across every mode—a within-brand deviation comparable in magnitude to the between-brand difference itself—despite being within its service life, under regular periodic maintenance and free of any recorded fault. As set out in the Results, neither ordinary device-to-device variation nor a spurious measurement accounts well for it, and its standby consumption was indistinguishable from that of its siblings, so the excess appeared only while the device was ventilating. An unidentified condition in the gas-delivery path—increased mechanical resistance, or a degraded blower or valve—is therefore the more plausible explanation, although we could not investigate it directly. The substantive point is that routine biomedical maintenance verifies clinical performance and safety but does not assess energy consumption, so a device may pass every scheduled check while drawing materially more power than an identical unit. If such a pattern proves general, periodic power measurement would be a low-cost addition to existing maintenance protocols, and the standby-to-active increment is a simple quantity for such a protocol to record.

The standby findings carry a separate and more immediately actionable implication. In none of the three brands did standby approach a genuine low-power state: the least favourable device family drew more than nine-tenths of its operating power while idle, and even the most efficient brand still drew over 40 W. Ventilators spend appreciable time between patient assignments, and in that interval the distinction between standby and switched off is, on these measurements, worth tens of watts per device continuously. Unlike procurement, this lever requires no capital expenditure and can be implemented immediately through unit-level protocols—consistent with the conclusion of Drinhaus and colleagues that the short start-up times of modern ventilators make powering down both safe and climate-beneficial (10).

### 4.2 Limitations

Several limitations temper these conclusions. First, power was measured with a consumer-grade plug-in meter rather than a laboratory-grade calibrated power analyser. The unit was calibrated by its manufacturer but received no additional calibration and was not independently verified against a reference instrument, so neither its calibration nor its stated ±2% accuracy is independently certified; inexpensive meters of this class typically perform less well at low loads than their headline specification implies. The meter does not report power factor, which we therefore did not record, so we cannot exclude the possibility that the three platforms differ in their electrical characteristics and that the meter’s accuracy differs correspondingly between them. Using a single meter unit for all measurements eliminates inter-meter variability but does not guarantee that measurement error is identical across devices of different design. What can be said is that the observed differences of 22 to 39 W exceed the meter’s stated uncertainty by more than an order of magnitude, so a device-dependent error would have to be implausibly large to generate a difference of this size. The use of a consumer-grade plug-in meter is also consistent with prior published measurements in this field (10).

Second, this was a single-centre study with three devices per brand, and its findings should be interpreted as proof-of-concept rather than as a definitive ranking of manufacturers. Although 81 active-mode observations were obtained, the independent experimental units are only nine ventilators, and the denominator degrees of freedom for the brand effect are correspondingly small at approximately six. The mixed-effects model was used in preference to comparing nine device means because the latter discards the within-device information entirely and, as the device-level Kruskal–Wallis analysis shows, lacks the resolution to detect the Brand B vs. Brand A difference at all; the model uses the repeated measurements to estimate the residual variance while correctly attributing the between-brand contrast to the device level. The parametric bootstrap was added precisely because the Satterthwaite approximation is unreliable at this sample size. The imprecision that remains is visible in the width of the emission intervals reported above. Multicentre replication with more devices per brand is required before these findings can support procurement policy.

Third, residual variance differed markedly between brands. Rather than proceeding under a violated homoscedasticity assumption, the primary model specifies a separate residual variance for each brand, which fitted substantially better and left every conclusion unchanged. The heterogeneity was driven principally by the single atypical Brand B device; excluding it reduced the Brand B standard deviation from 14.1 to 6.9 W. The concordance between the heteroscedastic model, the original homoscedastic specification, the parametric bootstrap, the assumption-free Kruskal–Wallis analysis and both sensitivity analyses provides reasonable assurance that the conclusions do not depend on the covariance structure adopted.

Fourth, because manufacturers offer different mode portfolios, the design was unbalanced with respect to ventilation mode: two harmonised categories were available on only two of the three brands, and the number of proprietary modes mapping to each category varied between brands from one to three. Only the synchronised intermittent mandatory ventilation category was represented equally across all three. The bounds we place on the mode effect are therefore firmer for categories represented across all three brands than for those that were not, and this is visible in the equivalence analysis, where volume control was the single category failing to meet the tighter ±5 W margin. The same imbalance means two interaction terms are not estimable, so the interaction test is complete only for the four categories common to all brands. A further caveat is that harmonisation groups modes by control logic rather than by internal implementation, so clinically equivalent modes may still be delivered by different engineering means with different electrical costs.

Fifth, measurements were made under controlled bench conditions rather than during live patient care. Real ventilation involves variable respiratory mechanics, patient effort, alarm conditions, suctioning and disconnection events, nebulisation and changes in ${\mathrm{FiO}}_{2}$, none of which were present here. Bench measurement was a deliberate choice, because it is the only way to hold ventilatory conditions identical across nine devices and thereby isolate the device as the source of variation, but it limits the extent to which the absolute values can be read as clinical consumption.

Two consequences of the load require separate treatment. Internal validity is preserved, because a single physical balloon and breathing circuit were used for every device, so whatever the load’s mechanical properties were, they were identical across brands. External reproducibility is genuinely limited: the balloon’s manufacturer and model were not recorded and its compliance and resistance were never characterised, so another centre cannot recreate the load and cannot expect to reproduce our absolute values, only the between-brand pattern. A calibrated lung simulator with defined mechanical properties would remove this obstacle and is recommended for future work.

Sixth, all modes for a given device were measured within a single session and in a fixed, non-randomised order. Testing for this directly showed a small negative trend of −0.45 W per sequence position, amounting to approximately 4 W across a device’s full mode sequence (1 to 7 W at the confidence limits), with no significant rank correlation within any individual brand. Because the order was fixed, this effect cannot be separated from a genuine mode effect. It is an order of magnitude smaller than the between-brand difference and was applied identically to every device, so it cannot generate that difference, but a small time-related or order-related drift cannot be excluded and randomisation of mode order is required in future work.

Seventh, spontaneous and supported modes were assessed without simulated inspiratory effort, so the values recorded for those modes reflect device behaviour under a passive load rather than during genuine patient-triggered support; this constraint applied equally to all three brands and is unlikely to have differentially affected the between-brand comparison, but it means the equivalence bounds placed on the mode effect cannot be extended to patient-triggered ventilation, and the likely direction of the omission is to understate consumption in supported modes.

Eighth, the standby measurements on which the baseline decomposition rests comprise a single reading per device, nine in total. The decomposition is therefore descriptive, carries no interval, and should be read as indicating where the between-brand difference resides rather than as a precise partition of it; confirmation would require repeated standby measurement alongside active measurement in a larger sample.

Ninth, the carbon estimates are scenario-based projections rather than measured quantities: they assume a given utilisation and a national average grid factor, and their confidence intervals propagate uncertainty in measured power alone. Actual utilisation, marginal rather than average grid intensity and uncertainty in the emission factor itself will all alter the absolute figures, though not the relative ranking of brands; Table 7 quantifies this dependence, across which the projected difference varies roughly tenfold. Only operational electricity consumption was assessed; manufacturing, transport and end-of-life emissions were not evaluated.

Finally, and most consequentially for the procurement question this study raises, all devices were approximately ten years old. While this made device age comparable across brands and thus strengthened the internal comparison, it limits external validity in precisely the respect most relevant to purchasing: the platforms measured here may no longer correspond to the models these manufacturers currently market, and power-supply architecture and gas-delivery technology have evolved over that interval. Our findings should therefore be read as demonstrating that clinically equivalent ventilators can differ substantially in energy consumption—a proposition that justifies measuring current models before purchase—rather than as a guide to which manufacturer should be selected today.

### 4.3 Future directions

Future work should extend these measurements to larger, multicentre samples of contemporary devices, employ calibrated reference instrumentation capable of recording true RMS power and power factor, use a lung simulator with defined mechanical properties, randomise the order in which modes are measured, and incorporate active inspiratory effort so that supported modes can be assessed under realistic conditions. Standby and active power should be recorded together and with equal care, so that the continuously energised baseline can be separated from the cost of ventilation itself; on our data that separation carried more of the between-brand difference than the ventilation increment did. Repeated measurement of the same devices over time would establish whether power consumption rises with device age, a possibility raised by the within-brand outlier observed here. Longitudinal measurement during genuine patient care would refine the emission estimates and support the development of formal energy-efficiency criteria for ventilator procurement, complementing emerging life-cycle approaches to medical-device sustainability (21).

### 4.4 Conclusions

Among three brands of ICU mechanical ventilator studied on the bench, brand—rather than ventilation mode—was the dominant measured determinant of electricity consumption, with the least efficient brand consuming approximately 22 to 39 W more than its comparators. Under continuous operation at the Turkish grid factor this projects to an additional 161 kg ${\mathrm{CO}}_{2}$-equivalent (95% CI 70–252) per ventilator per year, though the figure falls to 17 kg under a low-carbon grid and reduced utilisation, so the magnitude is assumption-dependent while the ordering is not. Every ventilation mode was equivalent within ±10 W under the conditions tested. The finding did not depend on a single atypical device. Standby was not a low-power state in any brand, reaching 92.6% of active consumption in one, and most of the difference between the highest- and lowest-consuming brands was already present with the devices idle.

Because a procurement penalty of this kind would be fixed at purchase and would persist for the service life of every device across a large installed base of continuously operating ventilators, we suggest that tenders for ICU ventilators should require measured power consumption to be declared and evaluated as a standardised criterion alongside clinical specifications, and that units adopt protocols for powering ventilators down rather than leaving them in standby. This is a recommendation about how devices should be assessed, not about which device to buy. As all devices studied were approximately ten years old and came from a single centre, these results establish that clinically equivalent ventilators can differ substantially in energy consumption, which is what justifies measuring current models before purchase; they do not identify which currently marketed manufacturer or platform should be preferred. Confirmation in larger, multicentre samples of contemporary devices is required before the magnitude reported here can be relied upon.

## Data Availability

The original contributions presented in the study are included in the article/Supplementary Material, further inquiries can be directed to the corresponding author.
