# Supplementary material for: Ventilator brand, not ventilation mode, determines electricity consumption and estimated carbon emissions: a bench study of nine intensive care ventilators
Source: Front Med (Lausanne). 2026 Oct 21;13:1966196. doi: 10.3389/fmed.2026.1966196 (PMC13731106; doi:10.3389/fmed.2026.1966196)
Supplement: Supplementary file 1 [file Datasheet3.pdf]

Data Sheet 3. Full output of the analysis script supplied as Data Sheet 2, run on the measurements supplied as Data Sheet 1. Every quantitative result reported in the article is reproduced below; each section heading gives the corresponding section or table of the article. Nothing in this file has been edited by hand.

```
====================================================================================
0.  SESSION INFORMATION AND DATA INTEGRITY
====================================================================================

Generated:        2026-09-04 01:03 +03
Input file:       Data_Sheet_1_ventilator_power_measurements.xlsx
Bootstrap seed:   20260904   (1000 simulations; 500 for the interaction)

R version 4.5.1 (2025-06-13 ucrt)
Platform:         x86_64-w64-mingw32
readxl:           1.5.0
nlme:             3.1.168
emmeans:          2.0.3

Observations read:           90
  active ventilation modes:   81
  standby:                    9
Devices:                      9  (3, 3, 3 per brand)
Harmonised mode categories:   6

Modes measured per device:

A1 A2 A3 B1 B2 B3 C1 C2 C3
 8  8  8 10 10 10  9  9  9

Brand-by-mode cells observed: 16 of 18  (two categories are unavailable by design)

          SIMV APRV/bilevel PRVC Pressure control (PC) Pressure support/CPAP Volume control (VC)
  Brand A    9           3    3                     3                     3                   3
  Brand B    9           3    0                     6                     6                   6
  Brand C    9           6    3                     3                     6                   0

====================================================================================
1.  DESCRIPTIVE FINDINGS, ACTIVE VENTILATION MODES
Article: Section 3.1; Table 1, upper panel
====================================================================================

    brand n_devices  n  mean    sd median  IQR  min   max
1 Brand A         3 24 64.95  6.34   65.8  2.8 36.3  69.4
2 Brand B         3 30 95.70 14.10   93.0 25.3 67.2 122.0
3 Brand C         3 27 55.87  2.89   55.4  2.7 52.8  66.3

Normality of the raw power values within brand (Shapiro-Wilk):
  Brand A   W = 0.459, p = <0.0001
  Brand B   W = 0.907, p = 0.0122
  Brand C   W = 0.810, p = 0.0002

Device-level mean active power (Figure 1):
  Brand A   A1 = 60.99   A2 = 67.86   A3 = 65.99
  Brand B   B1 = 85.81   B2 = 111.93   B3 = 89.35
  Brand C   C1 = 58.00   C2 = 53.46   C3 = 56.17

Highest-consuming device within Brand B: B2 (111.93 W)
  Brand B SD, all three devices:      14.1 W
  Brand B SD, B2 excluded:         6.9 W

Brand A active mean excluding the 36.3 W observation: 66.2 W
```

==

2. STANDBY CONSUMPTION

Article: Section 3.2; Table 1, lower panel

==

	brand	n	mean	sd	median	min	max	pct_of_active_mean
1	Brand A	3	60.13	5.11	57.7	56.7	66.0	92.6
2	Brand B	3	66.97	1.02	67.4	65.8	67.7	70.0
3	Brand C	3	40.73	1.56	40.9	39.1	42.2	72.9

Device-level Kruskal-Wallis across brands: H(2) = 6.49, p = 0.039

Lowest Brand B standby reading: 65.8 W

Brand C active measurements falling below it: 26 of 27

==

3. RESIDUAL VARIANCE STRUCTURE

Article: Section 2.6 and Section 3.3

==

Levene's test (median-centred) on active-mode power: F(2, 78) = 16.61, p = <0.0001

Model comparison, homoscedastic versus brand-specific residual variance (REML):

	Model	df	AIC	BIC	logLik	Test	L.Ratio	p-value
m_homo	1	10	526.4441	549.3487	-253.2220			
m_var	2	12	503.1592	530.6447	-239.5796	1 vs 2	27.28492	<.0001

The brand-heteroscedastic (varIdent) specification is adopted as the primary model.

==

4. PRIMARY ANALYSIS: BRAND-HETEROSCEDASTIC MIXED-EFFECTS MODEL

Article: Section 3.3; Table 2, primary model columns

==

Fixed effects, marginal (Type III) Wald F tests:

	numDF	denDF	F-value	p-value
(Intercept)	1	67	184.94765	<.0001
brand	2	6	18.43367	0.0027
mode	5	67	0.94896	0.4555

Residual standard deviation by brand:

Brand A 5.61 W

Brand B 8.13 W

Brand C 2.37 W

Device (random intercept) variance: 63.8

Intraclass correlation coefficient: 0.65

Estimated marginal means, averaged over harmonised mode category:

brand	emmean	SE	df	lower.CL	upper.CL
Brand A	64.9	4.76	6	53.2	76.5
Brand B	95.4	4.85	6	83.5	107.3
Brand C	56.3	4.66	6	44.9	67.7

Results are averaged over the levels of: mode

Degrees-of-freedom method: user-specified

Confidence level used: 0.95

Bonferroni-adjusted pairwise contrasts:

contrast	estimate	SE	df	t.ratio	p.value
Brand A - Brand B	-30.5	6.79	6	-4.496	0.0124
Brand A - Brand C	8.6	6.65	6	1.294	0.7301

contrast	estimate	SE	df	t.ratio	p.value
Brand B - Brand C	39.1	6.73	6	5.817	0.0034

Results are averaged over the levels of: mode
Degrees-of-freedom method: user-specified
P value adjustment: bonferroni method for 3 tests

Bonferroni-adjusted 95% confidence intervals for the same contrasts:

contrast	estimate	SE	df	lower.CL	upper.CL
Brand A - Brand B	-30.5	6.79	6	-52.9	-8.21
Brand A - Brand C	8.6	6.65	6	-13.3	30.45
Brand B - Brand C	39.1	6.73	6	17.0	61.25

Results are averaged over the levels of: mode
Degrees-of-freedom method: user-specified
Confidence level used: 0.95
Conf-level adjustment: bonferroni method for 3 estimates

==
5. HOMOSCEDASTIC SPECIFICATION (reported alongside the primary model)
Article: Section 3.3; Table 2, homoscedastic columns
==

Fixed effects, marginal (Type III) Wald F tests:

	numDF	denDF	F-value	p-value
(Intercept)	1	67	168.33627	<.0001
brand	2	6	17.12864	0.0033
mode	5	67	1.03975	0.4017

Residual standard deviation by brand:

Brand A	6.00 W
Brand B	6.00 W
Brand C	6.00 W

Device (random intercept) variance: 69.6
Intraclass correlation coefficient: 0.66

Estimated marginal means, averaged over harmonised mode category:

brand	emmean	SE	df	lower.CL	upper.CL
Brand A	64.8	4.98	6	52.6	76.9
Brand B	95.4	4.96	6	83.3	107.6
Brand C	56.4	4.97	6	44.2	68.6

Results are averaged over the levels of: mode
Degrees-of-freedom method: user-specified
Confidence level used: 0.95

Bonferroni-adjusted pairwise contrasts:

contrast	estimate	SE	df	t.ratio	p.value
Brand A - Brand B	-30.66	7.02	6	-4.368	0.0142
Brand A - Brand C	8.34	7.02	6	1.188	0.8393
Brand B - Brand C	39.00	7.02	6	5.556	0.0043

Results are averaged over the levels of: mode
Degrees-of-freedom method: user-specified
P value adjustment: bonferroni method for 3 tests

Bonferroni-adjusted 95% confidence intervals for the same contrasts:

contrast	estimate	SE	df	lower.CL	upper.CL
Brand A - Brand B	-30.66	7.02	6	-53.7	-7.58
Brand A - Brand C	8.34	7.02	6	-14.7	31.44
Brand B - Brand C	39.00	7.02	6	15.9	62.08

Results are averaged over the levels of: mode

Degrees-of-freedom method: user-specified
Confidence level used: 0.95
Conf-level adjustment: bonferroni method for 3 estimates

==

6. RESIDUAL DIAGNOSTICS
Article: Section 3.3

==

Shapiro-Wilk statistic on normalised residuals:
homoscedastic model W = 0.801, p = <0.0001
primary varIdent model W = 0.852, p = <0.0001
primary varIdent model, 36.3 W observation excluded W = 0.938, p = 0.0007

The improvement on excluding a single observation indicates that the residual non-normality is attributable to that reading rather than to the model.

==

7. PARAMETRIC BOOTSTRAP OF THE LIKELIHOOD-RATIO STATISTIC
Article: Sections 2.6 and 3.3

==

At nine devices the Satterthwaite approximation is unreliable, so the brand and mode effects are additionally tested by parametric bootstrap under the null model. Bootstrap p values are Monte Carlo estimates; the Monte Carlo standard error is given alongside each.

Brand effect:
likelihood-ratio statistic = 17.73 on 2 degrees of freedom
reference chi-square p = 0.0001
parametric bootstrap p = 0.003 (MC SE 0.002, 1000 successful simulations)

Ventilation mode effect:
likelihood-ratio statistic = 4.88 on 5 degrees of freedom
reference chi-square p = 0.4308
parametric bootstrap p = 0.509 (MC SE 0.016, 1000 successful simulations)

==

8. EQUIVALENCE ANALYSIS FOR VENTILATION MODE (two one-sided tests)
Article: Section 3.3; Table 5

==

Coefficients are relative to the reference category (SIMV), from the primary brand-heteroscedastic model, on 67 denominator degrees of freedom. Equivalence is established when the 90% confidence interval lies wholly inside the margin.

	mode_category	coefficient_W	CI90_lower	CI90_upper	TOST_p_10W	TOST_p_5W
1	APRV/bilevel	-2.13	-4.05	-0.21	<0.001	0.008
2	PRVC	-0.58	-2.99	1.83	<0.001	0.002
3	Pressure control (PC)	0.30	-1.99	2.59	<0.001	<0.001
4	Pressure support/CPAP	-0.70	-2.60	1.19	<0.001	<0.001
5	Volume control (VC)	1.62	-2.70	5.94	<0.001	0.098

==

9. BRAND-BY-MODE INTERACTION
Article: Section 3.3

==

Two brand-mode combinations are empty by design, so the interaction is tested as a cell-means model over the 16 observed combinations against the additive model.

```
  likelihood-ratio statistic  = 12.13 on 8 degrees of freedom
  reference chi-square p      = 0.1457
  parametric bootstrap p      = 0.267  (MC SE 0.020, 500 successful simulations)
```

Given nine devices and an unbalanced design this test has limited power. It should be read as finding no evidence of an interaction rather than establishing its absence.

==

10. MEASUREMENT-ORDER EFFECT

Article: Section 3.3 and Limitation 6

==

Modes were measured in a fixed, non-randomised order. Measurement_sequence gives the position of each mode within that device's single session (standby = 1).

Linear term for sequence position: -0.45 W per position (95% CI -0.80 to -0.10)
Likelihood-ratio test: 6.65 on 1 degree of freedom, p = 0.010

Spearman rank correlation between sequence position and power, within brand:

```
  Brand A   rho =  -0.12, p = 0.574
  Brand B   rho =  -0.03, p = 0.874
  Brand C   rho =  -0.17, p = 0.390
```

Because the order was fixed, sequence position and mode category are substantially confounded and cannot be separated in these data. The magnitude is an order of magnitude smaller than the between-brand difference and was applied identically to every device of every brand.

==

11. DEVICE-LEVEL KRUSKAL-WALLIS AND DUNN COMPARISONS

Article: Section 3.4

==

Device-level means (one value per ventilator, no distributional assumption):

	brand	device	power
1	Brand A	A1	60.98750
2	Brand A	A2	67.86250
3	Brand A	A3	65.98750
4	Brand B	B1	85.81000
5	Brand B	B2	111.93000
6	Brand B	B3	89.35000
7	Brand C	C1	58.00000
8	Brand C	C2	53.45556
9	Brand C	C3	56.16667

Kruskal-Wallis: H(2) = 7.20, p = 0.027; epsilon-squared = H/(n-1) = 0.90

Dunn post hoc comparisons (Bonferroni-adjusted):

	comparison	z	p_unadjusted	p_bonferroni
1	Brand A vs Brand B	-1.342	0.1797	0.5391
2	Brand A vs Brand C	1.342	0.1797	0.5391
3	Brand B vs Brand C	2.683	0.0073	0.0219

With three devices per group these rank-based tests have limited resolution; they corroborate the mixed model rather than replacing it.

==

12. SENSITIVITY ANALYSIS 1: SINGLE OUTLYING OBSERVATION EXCLUDED (a priori)

Article: Section 3.4.1

==

Excluded observation:

	brand	device	Proprietary_mode_name	mode	seq	power
8	Brand A	A1	APRV	APRV/bilevel	9	36.3

12a. Primary specification, 36.3 W observation excluded

Article: Section 3.4.1

Fixed effects, marginal (Type III) Wald F tests:

	numDF	denDF	F-value	p-value
(Intercept)	1	66	222.34103	<.0001
brand	2	6	20.10176	0.0022
mode	5	66	0.98787	0.4320

Residual standard deviation by brand:

Brand A 1.13 W
Brand B 8.28 W
Brand C 2.27 W

Device (random intercept) variance: 58.6

Intraclass correlation coefficient: 0.70

Estimated marginal means, averaged over harmonised mode category:

brand	emmean	SE	df	lower.CL	upper.CL
Brand A	66.1	4.43	6	55.2	76.9
Brand B	95.5	4.67	6	84.1	106.9
Brand C	55.9	4.45	6	45.0	66.8

Results are averaged over the levels of: mode
Degrees-of-freedom method: user-specified
Confidence level used: 0.95

Bonferroni-adjusted pairwise contrasts:

contrast	estimate	SE	df	t.ratio	p.value
Brand A - Brand B	-29.4	6.44	6	-4.573	0.0114
Brand A - Brand C	10.2	6.27	6	1.621	0.4682
Brand B - Brand C	39.6	6.45	6	6.141	0.0026

Results are averaged over the levels of: mode
Degrees-of-freedom method: user-specified
P value adjustment: bonferroni method for 3 tests

Bonferroni-adjusted 95% confidence intervals for the same contrasts:

contrast	estimate	SE	df	lower.CL	upper.CL
Brand A - Brand B	-29.4	6.44	6	-50.6	-8.27
Brand A - Brand C	10.2	6.27	6	-10.5	30.80
Brand B - Brand C	39.6	6.45	6	18.4	60.82

Results are averaged over the levels of: mode
Degrees-of-freedom method: user-specified
Confidence level used: 0.95
Conf-level adjustment: bonferroni method for 3 estimates

Excluding this reading reduces the fitted residual standard deviation for Brand A from 5.61 to 1.13 W. No conclusion is altered.

13. SENSITIVITY ANALYSIS 2: SINGLE OUTLYING DEVICE EXCLUDED (post hoc)

Article: Section 3.4.2; Table 6

This analysis was decided upon after inspection of the device-level means and forms

no part of the pre-specified analysis plan. It is reported as exploratory, alongside
and not in place of the primary model.

Excluded device: B2 (mean active power 111.9 W, versus 85.8 and 89.4 W for its two siblings)

==

13a. Primary specification, device excluded (5 denominator df)
Article: Section 3.4.2; Table 6, right-hand column

==

Fixed effects, marginal (Type III) Wald F tests:

	numDF	denDF	F-value	p-value
(Intercept)	1	58	1230.1393	<.0001
brand	2	5	66.1520	0.0003
mode	5	58	0.8605	0.5132

Residual standard deviation by brand:
Brand A 5.65 W
Brand B 6.95 W
Brand C 2.37 W
Device (random intercept) variance: 5.3
Intraclass correlation coefficient: 0.16

Estimated marginal means, averaged over harmonised mode category:

brand	emmean	SE	df	lower.CL	upper.CL
Brand A	64.8	1.77	5	60.3	69.4
Brand B	87.3	2.27	5	81.5	93.1
Brand C	56.1	1.48	5	52.3	59.9

Results are averaged over the levels of: mode
Degrees-of-freedom method: user-specified
Confidence level used: 0.95

Bonferroni-adjusted pairwise contrasts:

contrast	estimate	SE	df	t.ratio	p.value
Brand A - Brand B	-22.47	2.87	5	-7.830	0.0016
Brand A - Brand C	8.71	2.28	5	3.822	0.0370
Brand B - Brand C	31.18	2.71	5	11.501	0.0003

Results are averaged over the levels of: mode
Degrees-of-freedom method: user-specified
P value adjustment: bonferroni method for 3 tests

Bonferroni-adjusted 95% confidence intervals for the same contrasts:

contrast	estimate	SE	df	lower.CL	upper.CL
Brand A - Brand B	-22.47	2.87	5	-32.618	-12.3
Brand A - Brand C	8.71	2.28	5	0.657	16.8
Brand B - Brand C	31.18	2.71	5	21.601	40.8

Results are averaged over the levels of: mode
Degrees-of-freedom method: user-specified
Confidence level used: 0.95
Conf-level adjustment: bonferroni method for 3 estimates

Device variance component falls from 63.8 to 5.3; ICC from 0.65 to 0.16.
The between-brand effect persists and is more precisely estimated. The
Brand A versus Brand C comparison is exploratory in both columns and should not be
read as establishing a difference between those brands.

==

14. MODE-SPECIFIC POWER CONSUMPTION BY HARMONISED CATEGORY AND BRAND
Article: Table 4

==

	mode_category	Brand A	Brand B	Brand C
1	SIMV	66.0 +/- 2.0 (9)	96.5 +/- 13.1 (9)	56.3 +/- 4.0 (9)
2	APRV/bilevel	56.2 +/- 17.3 (3)	98.5 +/- 16.0 (3)	54.8 +/- 1.4 (6)
3	PRVC	65.4 +/- 1.0 (3)	n.a.	55.8 +/- 2.1 (3)
4	Pressure control (PC)	66.2 +/- 2.5 (3)	97.3 +/- 14.6 (6)	56.5 +/- 2.2 (3)
5	Pressure support/CPAP	67.4 +/- 2.0 (3)	89.3 +/- 16.1 (6)	56.1 +/- 3.2 (6)
6	Volume control (VC)	66.2 +/- 1.7 (3)	97.8 +/- 15.5 (6)	n.a.

Brand A, APRV/bilevel, excluding the 36.3 W observation: 66.2 +/- 1.4 (n = 2)

==

15. ESTIMATED ANNUAL ENERGY USE AND CO2-EQUIVALENT EMISSIONS

Article: Section 3.5; Tables 3 and 7

==

All figures below are scenario-based projections under stated assumptions, not measured quantities. Intervals propagate uncertainty in measured power only, and do not incorporate uncertainty in the emission factor or in device utilisation.

Continuous operation at the Turkish grid consumption-point factor (0.469 kg/kWh):

	brand	power_W	kWh_per_year	kg_CO2eq_per_year
1	Brand A	64.9 (53.2-76.5)	568 (466-670)	267 (219-314)
2	Brand B	95.4 (83.5-107.3)	836 (732-940)	392 (343-441)
3	Brand C	56.3 (44.9-67.7)	493 (393-593)	231 (184-278)

Between-brand differences expressed as annual energy and emissions:

	contrast	difference_W	kWh_per_year	kg_CO2eq_per_year
1	Brand A - Brand B	-30.5 (-52.9 to -8.2)	267 (72-463)	125 (34-217)
2	Brand A - Brand C	8.6 (-13.3 to 30.4)	not interpretable	not interpretable
3	Brand B - Brand C	39.1 (17.0 to 61.2)	343 (149-537)	161 (70-252)

Contrasts whose confidence interval includes zero are not converted to an emission difference, since the direction of the difference is not established.

Scenario sensitivity of the Brand B - Brand C difference (39.1 W), kg CO2-eq per ventilator per year:

	utilisation	0.469 (Turkiye)	0.440 (global average)	0.250 (EU average)	0.100 (low-carbon)
1	continuous (8760 h)	161 (70-252)	151 (66-236)	86 (37-134)	34 (15-54)
2	75% (6570 h)	121 (52-189)	113 (49-177)	64 (28-101)	26 (11-40)
3	50% (4380 h)	80 (35-126)	75 (33-118)	43 (19-67)	17 (7-27)

Across the range examined the projected difference varies from 17 to 161 kg.

Scaled to a 20-bed ICU at the Turkish grid factor:
  every bed ventilated continuously: 3.2 tonnes CO2-eq per year (1.4-5.0)
  75% ventilated-bed occupancy: 2.4 tonnes CO2-eq per year (1.0-3.8)

Proportional expression: the difference is 41.0% of Brand B consumption; equivalently Brand B consumed 69.5% more power than Brand C.

==

16. FULL SESSION INFORMATION

==

R version 4.5.1 (2025-06-13 ucrt)
Platform: x86_64-w64-mingw32/x64
Running under: Windows 11 x64 (build 26200)

Matrix products: default
  LAPACK version 3.12.1

```
locale:
[1] C

time zone: Europe/Istanbul
tzcode source: internal

attached base packages:
[1] stats     graphics  grDevices utils     datasets  methods   base

other attached packages:
[1] emmeans_2.0.3 nlme_3.1-168  readxl_1.5.0

loaded via a namespace (and not attached):
 [1] vctrs_0.7.3        cli_3.6.5          rlang_1.2.0        TH.data_1.1-4      estimability_1.5.1
 [6] xtable_1.8-4       zoo_1.8-14         glue_1.8.0         grid_4.5.1         cellranger_1.1.0
[11] tibble_3.3.0       MASS_7.3-65        mvtnorm_1.3-3      lifecycle_1.0.5    compiler_4.5.1
[16] multcomp_1.4-28    codetools_0.2-20   sandwich_3.1-1     coda_0.19-4.1      pkgconfig_2.0.3
[21] rstudioapi_0.17.1  lattice_0.22-7     pillar_1.11.0      splines_4.5.1      magrittr_2.0.5
[26] Matrix_1.7-3       tools_4.5.1        survival_3.8-9
```
